# Disruptions in Cognitive‐Affective Circuitry in Major Depression Disorder: Insights From REST‐Meta‐MDD and Its Implication for Predicting TMS Treatment Efficacy

**DOI:** 10.1111/cns.70533

**Published:** 2025-08-04

**Authors:** Na Zhao, Liang Li, Matthew Lock, Yi‐Fan Ai, Jian Liu, Chun‐Ying Zhu, Yu‐Feng Zang, Hua‐Ning Wang, Bao‐Juan Li

**Affiliations:** ^1^ Center for Cognition and Brain Disorders/Department of Neurology The Affiliated Hospital of Hangzhou Normal University Zhejiang Hangzhou China; ^2^ Institute of Psychological Sciences, Hangzhou Normal University Zhejiang Hangzhou China; ^3^ Zhejiang Key Laboratory for Research in Assessment of Cognitive Impairments Zhejiang Hangzhou China; ^4^ The Brain Science Center, Beijing Institute of Basic Medical Sciences Beijing China; ^5^ Center for Psychological Sciences, Zhejiang University Hangzhou China; ^6^ Teaching and Research Support Center, Air Force Medical University Xi'an Shaanxi China; ^7^ The Third Affiliated Hospital of AFMU, Air Force Medical University Xi'an Shaanxi China; ^8^ The Department of Psychology The Affiliated Hospital of Hangzhou Normal University Zhejiang China; ^9^ Department of Psychiatry Xijing Hospital, Air Force Medical University Xi'an China; ^10^ School of Biomedical Engineering, Air Force Medical University Xi'an Shaanxi China

**Keywords:** amygdala, anterior cingulate cortex, cognitive‐affective circuitry, effective connectivity, major depressive disorder, predicative power, spectral dynamic causal model

## Abstract

**Aims:**

Major depressive disorder (MDD) is a common psychiatric disorder whose causes and manifestations are diverse and numerous. To facilitate targeted therapeutic interventions, we characterized the abnormalities in effective connectivity within the cognitive–affective (CCN—AN) circuits to identify predictive biomarkers of TMS efficacy based on a large multicenter dataset and an independent dataset from patients receiving TMS.

**Methods:**

Both functional and effective connectivity (FC, EC) were analyzed. As there was only one significant connection observed in FC, classification based on the differences in EC was performed using REST‐meta‐MDD. Furthermore, correlations between these abnormal connectivity and depression severity, as well as depression and suicidality alleviation, were calculated to determine their predictive implications for TMS efficacy using an independent dataset.

**Results:**

Overall increased connectivity from the AN to the CCN and decreased connectivity from the CCN to the AN in MDD were observed using EC. These disruptions drove the classification accuracy up to 79.1%. Furthermore, the connection from the right inferior parietal lobule (IPL. R) to the right amygdala (AMYG.R) was negatively correlated with depression scores. Notably, the IPL connectivity to the anterior cingulate cortex (ACC) and the AMYG.R were closely correlated with depression and suicidal ideation alleviation following TMS treatment.

**Conclusions:**

These findings suggest that MDD is characterized by disruptions in both top‐down and bottom‐up emotion regulation systems. Notably, the key abnormal connectivities, particularly those from the IPL to ACC and AMYG, could predict the efficacy of TMS treatment. This insight refines MDD diagnosis and paves the way for more precise targeted interventions in the future.

## Graphical abstract

Disruptions of the cognitive‐affective circuitry, involving both top‐down and bottom‐up emotion regulation systems, in MDD were reliably detected across multiple centers. These key anomalies, particularly the abnormal connections from the IPL to ACC and AMYG, could predict TMS treatment efficacy.

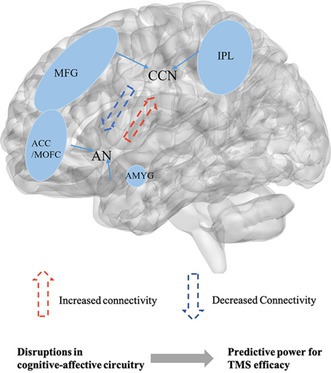

## 1 Introduction

Major depression disorder (MDD) is a severe and complex psychiatric disease characterized by depressed mood, waning interest, and cognitive debilitation, along with an array of autonomic manifestations, which exist in a spectrum of variations among individuals [1, 2]. Presently, the diagnosis of MDD rests upon phenomenological symptoms discovered through scales performed by psychiatrists during clinical assessments in the hospital. This approach, reliant on subjective self‐reporting, remains susceptible to influences stemming from both the psychiatrist and patients themselves, causing a plethora of issues that impede both a timely and accurate diagnosis, which eventually delays an effective treatment [3, 4]. We hope that identifying biomarkers of MDD—and its response to treatment—may facilitate targeted therapeutic interventions.

Considerable research supports the existence of disruptions of MDD within a widespread network, rather than abnormalities in individual brain areas [5, 6, 7, 8]. It has even been postulated that MDD involves a breakdown of the coordination between the cognitive control network (CCN) and the affective network (AN) [9, 10]. Numerous studies have illuminated the importance of cognitive and emotion processing in MDD, underpinned by extensive abnormalities within the CCN and AN [5, 6, 7, 11]. Moreover, retrospective studies found that antidepression treatments, such as antidepressant medicine [12], cognitive behavior therapy (CBT) [13], Electroconvulsive Therapy (ECT) [14], and especially transcranial magnetic stimulation (TMS) [15, 16], took effect by altering the connectivity and interactions between the CCN and AN. These connection alterations are important biomarkers for subsequent therapies.

As a treatment, high frequency‐TMS over the DLPFC is a non‐invasive modulation technique and supported by Level A evidence from the International Federation of Clinical Neurophysiology, demonstrating robust antidepressant efficacy and recommended for patients unresponsive to first‐line pharmacotherapies [17, 18]. Moreover, the FDA has endorsed the clinical application of functional magnetic resonance imaging (fMRI)‐guided TMS, that is, Stanford Neuromodulation Therapy (SNT), for treatment‐refractory depression [19]. This enables precise circuit‐based intervention and investigation of underlying neural mechanisms. Even though TMS offers rapid relief for MDD symptoms including depression and suicidal ideation [20, 21], the underlying neural mechanism of its therapeutic effects is largely unknown. Therefore, it is of great importance to identify the core anomalies in the CCN‐AN co‐network complex that reflect MDD pathology, and explore its predictive implications for individualized neuronavigational TMS treatment efficacy. This line of study promises to refine our understanding of MDD and inform future targeted interventions for optimizing patient care and perhaps help redefine how we diagnose and assess MDD in the future.

Despite extensive research into the atypical correlations of the whole brain [5, 6, 7, 8, 22, 23], studies specifically targeting the co‐network complex of the CCN‐AN remain scarce. Moreover, small sample sizes and data collection parameter variations resulted in large heterogeneity among previous studies. Collectively, these studies underscore the need to investigate the pathophysiology of MDD by probing the complex relationship connecting the CCN and AN. Such exploration promises to enhance the identification of objective biomarkers for MDD, thereby advancing diagnostic accuracy and enabling swift, precisely targeted interventions.

Additionally, these previous studies predominantly centered on conventional functional connectivity (FC), which is useful for illustrating interactions among brain regions but does not provide causal insights. In contrast, effective connectivity (EC), based on the spectral dynamic causal model (spDCM), delineates not only the influences exerted by one neural system upon another, but also reveals the directionality of signals within or between networks [24]. This approach not only demonstrates superior discriminatory capacity between MDD and healthy controls (HC) [25], but also boasts applicability as an efficacious biomarker for MDD diagnosis [25, 26, 27] and a tool for unraveling underlying mechanisms that might eventually lead to more targeted therapy for MDD [28, 29].

Consequently, we now endeavor to unravel the abnormalities in connectivity within MDD patients, focusing on the relationship between the CCN and AN systematically. These irregular connections existed within the CCN‐AN and were utilized as discriminatory features for distinguishing MDD patients from healthy controls, with the goal of identifying a pivotal biomarker of MDD that would enhance MDD diagnosis. Moreover, the extent to which reported clinical severity is reflected by these disruptions of CCN‐AN circuits is largely unknown. To address this problem, we assessed the correlation between the connectivity strengths and clinical scores. Additionally, the predictive validity of these abnormal connections was then assessed using an independent dataset acquired before transcranial magnetic stimulation (TMS) treatment. Specifically, we asked whether the baseline (pre‐TMS) effective connectivity (EC)—in connectivity showing differences between patients and HC—could predict treatment responses to TMS in an independent cohort of patients. This endeavor holds the potential to profoundly augment our comprehension of the cognitive and emotional regulation neural framework in MDD, based on a large multi‐center dataset that helps to overcome the small sample limitation in previous studies.

In this report, we focus on EC but also report a complementary analysis of FC for comparison. We focus on EC because EC estimates are based upon FC measured with complex cross spectra or coherence in the frequency domain. In other words, functional connectivity (i.e., correlations or coherence) is the result of directed effective connectivity.

Crucially, changes in a small number of effective connectivity can produce widespread changes in functional connectivity throughout a network. We therefore anticipated (and confirmed) that the differences in EC would have greater discriminatory power in differentiating between healthy controls and patients—and greater predictive ability in relation to treatment responses.

Finally, it should be noted that EC is estimated for recurrent or self‐connectivity. This is important because self‐connectivity reflects differences in neuromodulatory gain (e.g., excitation and inhibition balance) that are often associated with the pathophysiology of psychiatric disorders. These differences in (intrinsic) connectivity cannot be measured with functional connectivity, which is restricted to between‐region measures.

## 2 Materials and Methods

### 2.1 Datasets and Preprocessing

Two datasets (Dataset‐1 and Dataset‐2) were included in this study. The Dataset‐1 was obtained from the REST‐meta‐MDD project initiated in China (http://rfmri.org/REST‐meta‐MDD). In total, 1642 subjects (MDD vs. HC: 848 vs. 794) from 17 sites were included in this study after inclusion and exclusion criteria according to previous research [30]. Detailed information regarding the imaging acquisition and data preprocessing was illustrated in said study and can be referred to for further inquiry [30].

**FIGURE 1:**
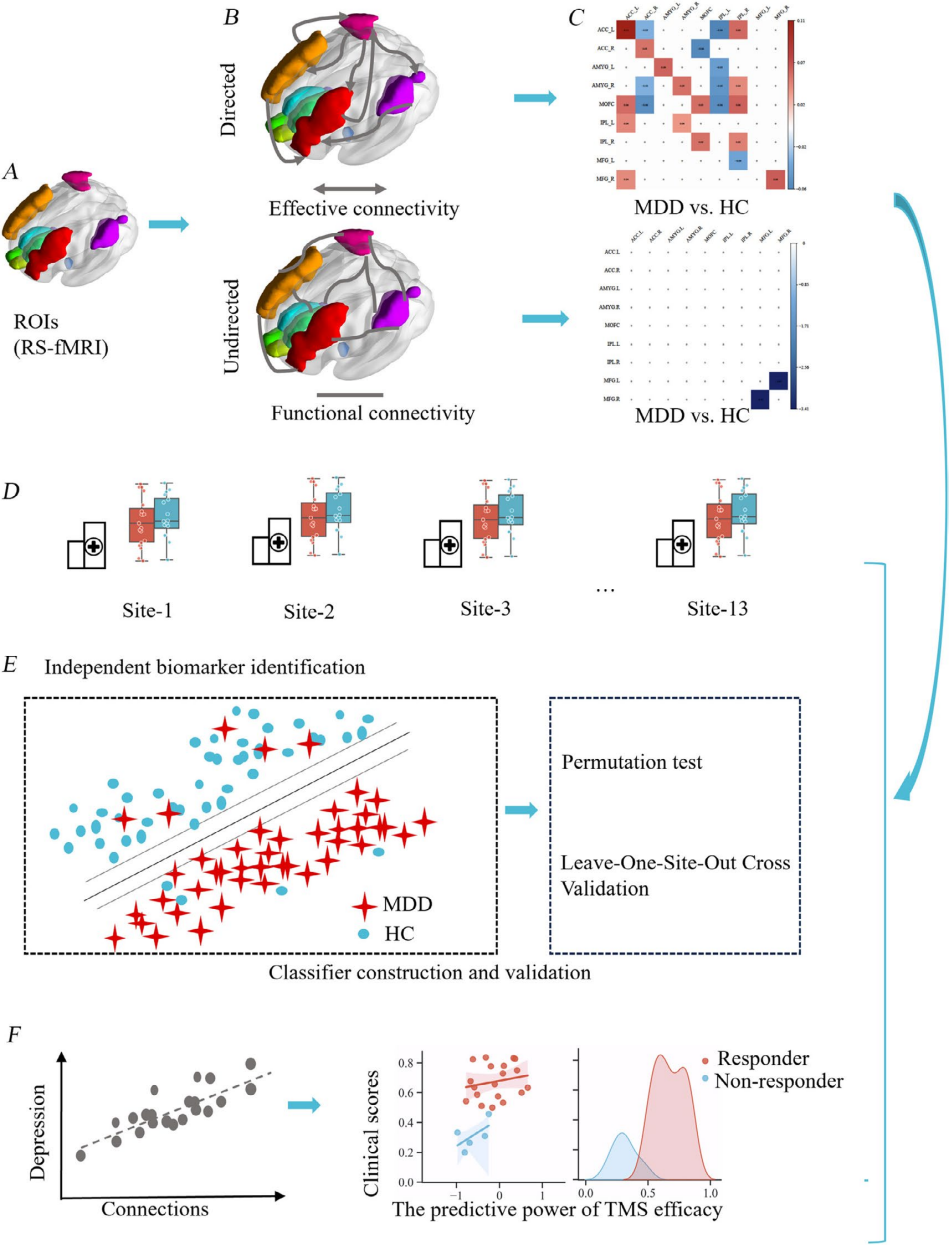
The data analysis flowchart of the whole study. (A) ROIs selection; (B) ROI‐wise effective and functional connectivity were calculated; (C) Significant differences in effective and functional connectivity between MDD and HC; positive value represent higher connectivity in MDD compared with HC, while negative value represent lower connectivity in MDD compared with HC; as only one connection existed significant differences between MDD and HC (FDR correction, *q* < 0.05), only effective connectivity (EC) was included into subsequent analysis (D, E, F); D: Comparisons of measures for each site between MDD (red) and HC (blue); (E) Identifying the abnormalizes and constructing an objective biomarker for MDD (MDD: Red star; HC: Blue circle); (F) Correlations between the aberrant connectivity and depression scores, and their predictive implications for the effectiveness of TMS therapy.

The other independent dataset (Dataset‐2), which consists of 28 MDD patients with suicidal ideation, was treated with Stanford Neuromodulation Therapy (SNT), that is, an individualized neuronavigational TMS therapy. This data was utilized to validate the implications of the abnormal connectivity obtained from Dataset‐1 for TMS treatment efficacy. We performed data preprocessing using the same procedure as Dataset‐1. All data analysis flowcharts for the study are displayed in Figure 1.

### 2.2 ROI Selection

A total of 9 ROIs (Table 1, Figure S1) within the CCN and AN were included in the subsequent FC and EC analyses to explore the connectivity between CCN and AN. These ROIs were selected based on previous research [31, 32, 33, 34]. Among them, the ROIs contained within the CCN were selected according to Yeo et al. [31], while those in the AN were selected according to Kaiser et al. [5] and Pizzagalli et al. [35]. All of these ROIs were defined according to the corresponding brain regions in the Automated Anatomical Labeling (AAL) atlas. In addition, the left and right medial orbitofrontal cortex (OFC) were combined as the middle medial orbitofrontal cortex (MOFC), respectively.

**TABLE 1:** The ROIs within CNN and AN included in spDCM.

Network	ROIs	MNI coordinates			ROI signal no.
Cognitive control network (CCN)	Left middle frontal gyrus (MFG.L)	−34	33	35	7
	Right middle frontal gyrus (MFG.R)	37	33	34	8
	Left inferior parietal lobule (IPL.L)	−44	−46	47	61
	Right inferior parietal lobule (IPL.R)	45	−46	50	62
Affective network (AN)	Left anterior cingulate cortex (ACC.L)	−5	35	14	31
	Right anterior cingulate cortex (ACC.R)	7	37	16	32
	Left amygdala (AMYG.L)	−24	−1	−17	41
	Right amygdala (AMYG.R)	26	−1	−18	42
	Medial orbitofrontal cortex (MOFC)	0	52	−7	25, 26

*Note:* The ROI Signal No. corresponds to the column number of the ROI Signals in the REST‐meta‐MDD.

### 2.3 Effective Connectivity Based on spDCM

For each subject, to explore the interactions between CCN and AN, all ROIs were included to construct a single model based on spectral dynamic causal modeling (spDCM) using SPM12 (https://www.fil.ion.ucl.ac.uk/spm/software/spm12/). This process generated subject‐specific connectivity parameters that quantified the connection strengths within these networks. Specifically, ComBat (https://github.com/Jfortin1/ComBatHarmonization) [36, 37] was used to remove influences due to variation in collection parameters across sites upon the constructed single model. Subsequently, these parameters (subject‐specific connection strengths) from the first level were entered into a second‐level analysis employing a Parametric Empirical Bayes (PEB) model within a General Linear Model (GLM) framework. The GLM was designed to investigate both shared and differential connectivity patterns between patient and healthy control groups. Specifically, the first covariate of the design matrix was set to 1 to capture common connectivity features across groups, while the second covariate, with values of 1 for controls and −1 for patients, modeled group differences.

In optimizing the PEB model, its evidence was compared against that of reduced models where some regression parameters were turned off. This step aimed to identify the necessity of individual parameters. A Bayesian Model Average (BMA) was then conducted over all the reduced models to derive averaged parameters weighted by their respective model probabilities. Notably, demographic variables including age, gender, and education level were regressed out during the GLM stage to isolate network connectivity features from these factors [38, 39].

Moreover, the EC was averaged within brain regions of both the AN and CCN, and connectivity between brain regions belonging to these two networks was analyzed using a conventional linear model to observe the overall discrepancies between MDD and HC.

### 2.4 Functional Connectivity

For each ROI, the averaged time series were extracted, then the ROI‐wise functional connectivity, that is, the Pearson's correlation, between each pair of time series was calculated. The generalized linear model was analyzed to evaluate the FC differences between MDD and HC while regressing out age, gender, and education.

### 2.5 Dependable Biomarkers Observed in the CCN‐AN Circuitry

In pursuit of a discerning biomarker for MDD patients, we created a classification model using a supporting vector machine (SVM). The dataset was divided into a training dataset and a testing dataset in a 4:1 ratio to conduct two distinct phases: training and testing. Here, only one connection measured by FC showed a significant difference after FDR correction (*q* < 0.05). Then, we conducted the SVM analysis using the EC measure. Initially, the EC within the ROIs had a 95% probability of discrepancies between MDD and HC, which was derived from the PEB analysis. This was then employed as a representative feature and put into the SVM model to classify the MDD patients and HC. The parameters of a generative model (e.g., a dynamic causal model) to discriminate between groups or classify patients are known as generative embedding [40]. Generative embedding rests on the assumption that the key parameters of a model that generates data features, such as functional connectivity, have greater discriminatory power than the data features they generate. In our case, the parameters correspond to the effective connectivity within and between regions. Subsequently, the grid search method with five‐fold cross‐validation (CV = 5) was used to facilitate the identification of the optimal classification (SVM) parameters, including c (penalty coefficient) and g (gamma for kernel function). Upon identifying c (1e+05) and g (1e‐05), the optimal hyperplane was derived from the training dataset. This hyperplane was then applied to the remaining testing dataset to estimate its classification efficacy. Meanwhile, sensitivity, specificity, and the receiver operating characteristic (ROC) curve's area under the curve (AUC) were also employed to quantify the predictive performance of the established classification model.

Furthermore, we assessed the model's classification performance through a permutation test. In this process, the labels of MDD and HC were randomized across 1000 permutations while retaining the original features, generating a null probability distribution of classification. This distribution under the null hypothesis suggests that there is no dependency between the features and labels. Subsequently, we calculated an empirical *p*‐value as a percentage of all permutations where the randomized label accuracy exceeded that of the unpermuted original testing dataset.

Additionally, the generalization capability of the established model was also assessed using Leave‐One‐Site‐Out Cross Validation (LOSOCV). For this analysis, we excluded sites that were recruited for less than 20 subjects; in the end, only 13 sites were included in the LOSOCV. Figure S2 can be referred to for the demographic and head motion information regarding the 13 sites.

### 2.6 The Correlation Between Effectivity Strength and Clinical Scores

To investigate the extent to which the MDD group is reflected by the disruptions of CCN‐AN circuits, Pearson's correlations were conducted between the connection (both EC and FC) measures and the Hamilton Depression Rating (HAMD) scores.

### 2.7 The Predictive Power of TMS

To estimate whether the abnormal connectivity could predict TMS treatment outcome, an independent dataset (Dataset‐2), which consists of MDD patients treated with Stanford Neuromodulation Therapy (SNT), was included. From this dataset, 28 patients with MDD completed both pre‐ and post‐treatment MRI scanning (detailed information can be found in our previous research [20]). For contrast, 25 healthy subjects were included. After preprocessing, only 26 patients with MDD were included in the subsequent DCM analysis due to excessive head motion. We then extracted the ECs of the dataset in the same fashion as done previously for the REST‐meta‐MDD dataset, which showed differences between MDD and HC with 95% probability (*p* > 0.95). Notably, *p* > 0.95 in the PEB framework represents at least a 95% posterior probability of the directional effects (i.e., the EC) differing from those of HC, which is consistent with the classical hypothesized effect (*p* < 0.05). Because one MDD patient's pre‐treatment scanning was not convergent for the DCM analysis, a final total of 25 MDD patients' pre‐treatment data were included. To estimate whether the baseline effective connectivity could predict TMS efficacy, we calculated Pearson's correlation between the ECs and clinical scores, including HAMD‐17 scores, Montgomery‐Asberg Depression Rating Scale (MADRS) scores, and Beck Suicide Intentionality Scale—Chinese Version (BSI‐CV) scores.

## 3 Results

### 3.1 Basic Information of Included Subjects

Ultimately, due to non‐convergence in the spDCM analysis and the influence of outliers, 908 (442 patients with MDD and 468 HCs) subjects were included in the group analysis. The detailed demographic information of these subjects can be found in Table 2 and Figure S2. Overall, no significant differences between MDD and HC in regard to age, gender, and head motion, except that MDD's education displayed lower than that of HCs (*p* < 0.001). After removing the sites with less than 20 subjects, significant differences in education between MDD and HC were observed in S1, S3, S4, S5, S12, and S13. Furthermore, among these subjects, only 395 subjects had HAMD scores.

**TABLE 2:** The basic information of included participants.

	MDD (*N* = 468)	HC (*N* = 440)	Statistics
Sex (male/female)	176/292	191/249	*χ* ^*2*^ = 3.17, *p* = 0.075
Age (mean ± SD)	34.7 ± 11.5	33.6 ± 12.3	*t* = 1.40, *p* = 0.092
Education (mean ± SD)**	11.99 ± 3.56	13.77 ± 3.49	*t = −7.62, p < 0.001*
mFD (mean ± SD)	0.06 ± 0.04	0.07 ± 0.04	*t* = −1.18, *p* = 0.63

Abbreviations: mFD, mean Frame displacement; SD, standard division.

** *p* < 0.01.

### 3.2 Disruptions in CCN‐AN Circuitry for MDD

**FIGURE 2:**
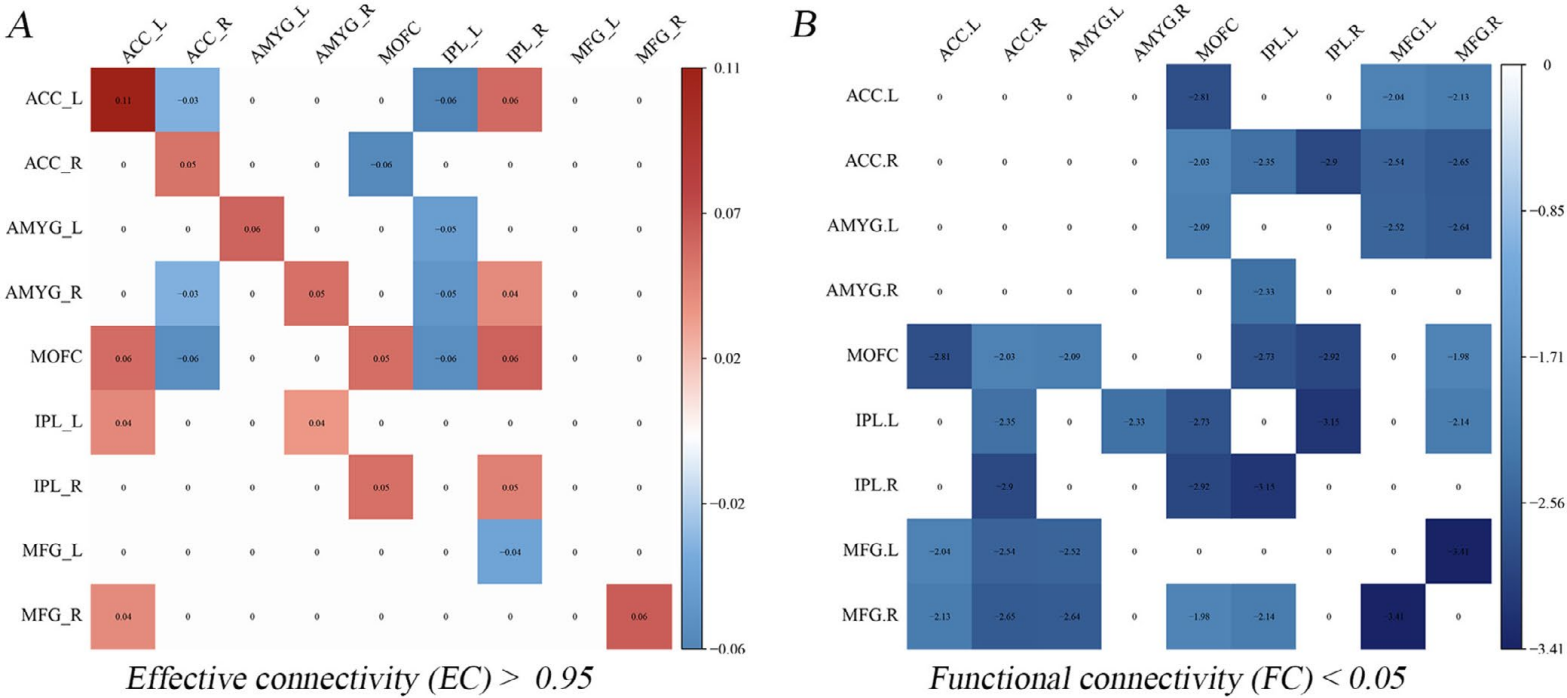
The discrepancies in effective connectivity (EC) and functional connectivity (FC) between MDD and HC groups obtained from Dataset‐1. (A) Differences in EC between MDD and HC detected by Parametric Empirical Bayes (PEB) analysis within a general linear model (GLM) framework (*p* > 0.95), with demographic information (age, gender and education) statistically controlled; (B) Differences in FC between MDD and HC detected using a general linear regression model (*p* < 0.05) with similarly accounted for age, gender, and education as nuisance variables.

Generally, regardless of whether they were from the MDD or HC group, excitatory effects were observed within the AN and CCN, while connectivity between the two networks was inhibitory (i.e., negative). With the AN, compared to HC, MDD patients displayed increased self‐connectivity of the bilateral anterior cingulate cortex (ACC), amygdala (AMYG), MOFC, and the connectivity from the left ACC (ACC.L) to MOFC, while decreased influence from the right ACC (ACC.R) to the right AMYG (AMYG.R) and MOFC, and from the MOFC to the ACC.R (Figure 2A). For the CCN, MDD showed higher self‐connectivity of the right inferior parietal lobule (IPL.R) and right middle frontal gyrus (MFG.R), whereas there was reduced influence from the IPL.R to the left MFG (MFG.L) (Figure 2A).

Significant connectivity was observed between CCN and AN (Figure S3). Compared to HC, MDD patients observed slightly increased connectivity from AN to CCN, while simultaneously observing decreased connectivity from CCN to AN (Figure S3). Specifically, the IPL.L induced inhibitory effects on the ACC.L, AMYG.L, AMYG.R, MOFC. While the connectivity from IPL.R to ACC.R, AMYG.R and MOFC increased (Figure 2A). Similarly, excitatory effects were observed from the AN to the CCN, with ACC.L increasing in connectivity to the IPL.L, MFG.R, AMYG.R to IPL.L, and MOFC to IPL.R (Figure 2A).

Strongly differing from EC, MDD patients displayed overall reduced FC for all brain areas (*p* < 0.05, uncorrected, Figure 2B), with only the connectivity between the MFG.L and MFG.R significantly decreased (FDR correction, *q* < 0.05, Figure S4).

For all included sites with more than 20 subjects, there were no significant differences in either the AN or CCN ECs between MDD and HC (Figure S5A,B). However, significantly increased connectivity was observed from AN *to* CCN at 4 sites (Figure S5C). Generally, there were no significant differences in connectivity from the CCN to AN between MDD and HC, except one location in which MDD showed significantly higher connection compared to HC (Figure S5D).

### 3.3 Objective Biomarkers Observed in the CCN‐AN Circuitry

**FIGURE 3:**
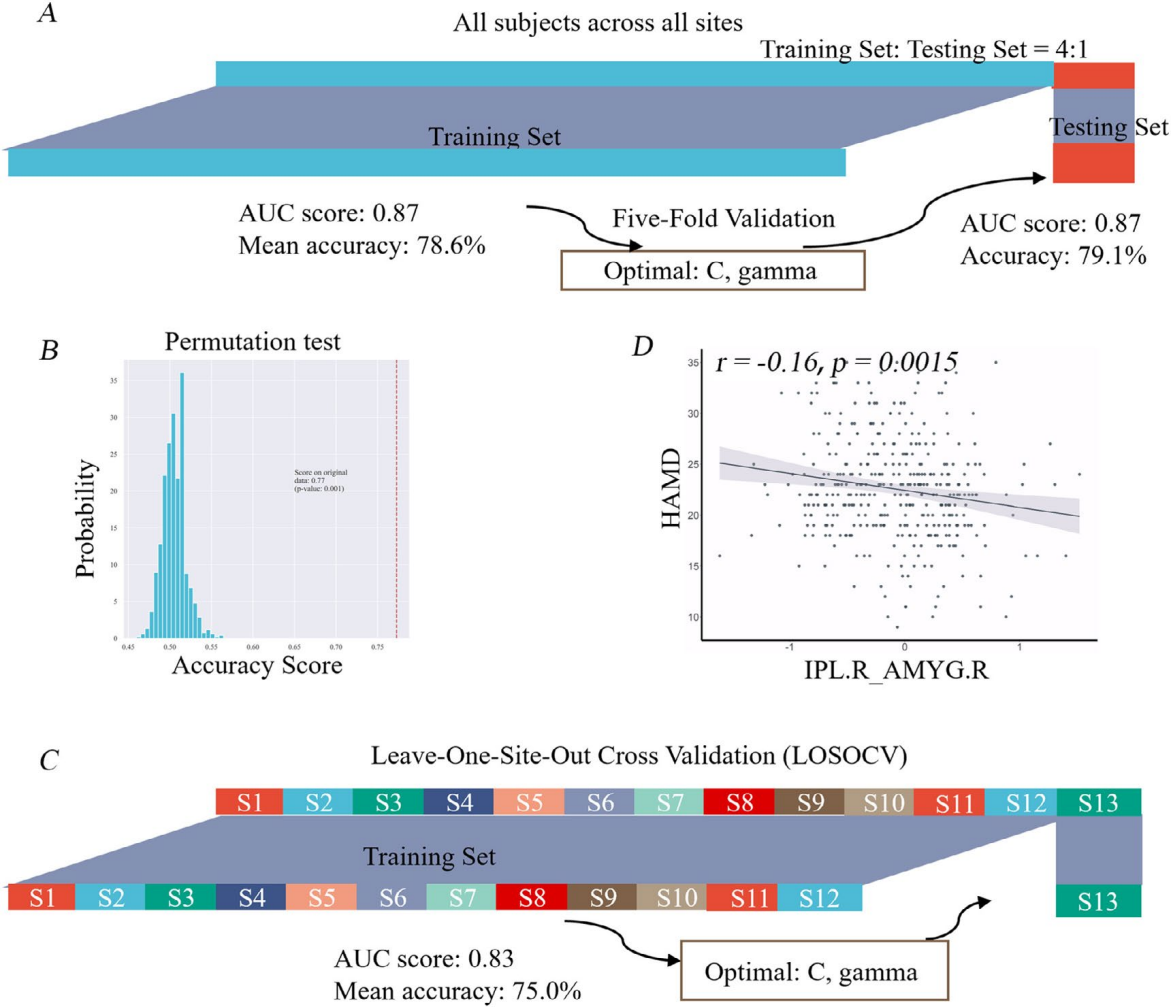
The classification model and the correlations between effective connectivity (EC) with depression scores. (A) The classifier based on all subjects across all 17 sites; the accuracy is 79.1%. (B) A null probability distribution of classification accuracy with the randomized labels generated through 1000 permutations. (C) The classifier was validated using leave‐one‐site‐out cross validation (LOSOCV), the accuracy score was 75%. (D) The Pearson's correlation between the baseline effective connectivity from the IPL.R to the AMYG.R and Hamilton Depression Rating Scale (HAMD) score. AMYG.R: Right amygdala; AUC, Area under the curve; IPL.R, Right inferior lobule.

A total of 24 edges were included in our classification model. Through five‐fold cross‐validation with parameter optimization (detailed classification performance outcomes in Table S1), the model demonstrated robust discriminative capacity with an AUC value of 0.87 (Figure 3A). Permutation testing confirmed the model's statistical significance in distinguishing MDD from HC groups (Figure 3B; *p* < 0.001 with randomized labels). The classifier displayed an accuracy rate of 79.1%, with a specificity rate of 78.7%, and a sensitivity rate of 79.6%.

The classifier's performance was also assessed using LOSOCV (Figure 3C). For each site, the AUC, specificity, sensitivity, and classification accuracy are displayed in Table S2. The mean AUC for all sites was 83% (SD: 7.77%) (Figure 3C).

### 3.4 The Connection Strength Correlated With Depression Severity

Regarding the circuit of CCN‐AN, the IPL.R to AMYG.R connectivity was found to be negatively correlated with the depression severity (Figure 3D). Moreover, the higher self‐connectivity of the ACC (ACC.L, ACC.R), AMYG (AMYG.L, AMYG.R), MOFC, IPL.R, and MFG.R was positively correlated with HAMD scores (Figure S6). In dynamic causal modeling, self‐connectivity is modeled as inhibitory. Therefore, a stronger self‐connectivity means a greater self‐inhibition, which models a reduced gain or responsiveness to extrinsic stimuli [24, 41].

Finally, we did not observe significant correlations between the conventional FC and depression scores.

### 3.5 The Implications of Abnormal Connectivity for Predicting TMS Treatment Efficacy

**FIGURE 4:**
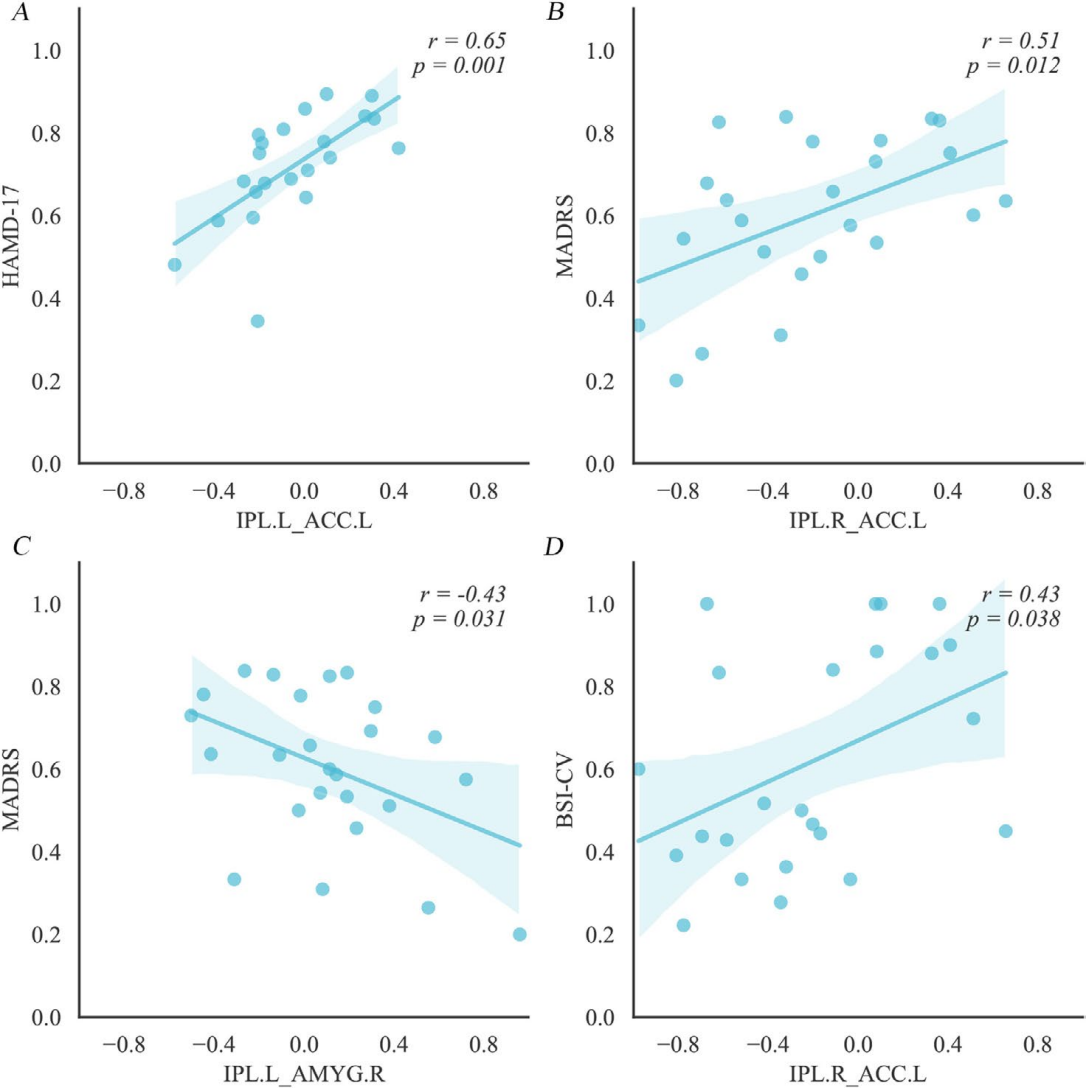
The predictive power of the abnormal connectivity within the affective network (AN) and cognitive control network (CCN) circuits for TMS antidepressant and anti‐suicidality efficacy. (A) The Pearson's correlations (*r* = 0.65, *p* = 0.001) between the effective connectivity from the IPL.L to ACC.L and HAMD‐17 reduction scores after TMS treatment for MDD patients; (B) The Pearson's correlation (*r* = 0.51, *p* = 0.012) between the effective connectivity from the IPL.R to ACC.L and MADRS reduction after TMS treatment for MDD patients; (C) The Pearson's correlation (*r* = −0.43, *p* = 0.031) between the effective connectivity from the IPL.L to AMYG.R and MADRS reduction after TMS treatment for MDD patients; (D) The correlation (*r* = 0.43, *p* = 0.038) between the effective connectivity from the IPL.R to ACC.L and BSI‐CV reductions; HAMD‐17, 17‐item Hamilton Depression Rating Scale; MADRS, Montgomery‐Asberg Depression Rating Scale; BSI‐CV, Beck Suicide Intentionality Scale—Chinese Version; IPL.L, The left inferior parietal lobule; IPL.R, The right inferior parietal lobule; ACC.L, The left anterior cingulate cortex; AMYG.L, The left amygdala gyrus.

Abnormal connectivity between the CCN and AN predicted TMS treatment efficacy. Baseline EC from the IPL to the ACC.L was positively correlated with depression reduction (*r* = 0.65, *p* = 0.001; *r* = 0.51, *p* = 0.012; as measured by HAMD‐17 and MADRS) following TMS therapy (Figure 4A,B). Conversely, EC from the IPL.L to the AMYG.R was negatively correlated with MADRS reduction (*r* = −0.43, *p* = 0.031; Figure 4C). Moreover, the abnormal connectivity of IPL.R to ACC.L was strongly linked to reductions in BSI‐CV scores (*r* = 0.43, *p* = 0.038; Figure 4D).

The self‐connectivity of bilateral AMYG and MFG.R were positively correlated with reductions in depression scores. Specifically, the self‐connectivity of the AMYG.L showed close associations with decreases in both HAMD‐17 and MADRS scores (*r* = 0.48, *p* = 0.020; *r* = 0.51, *p* = 0.012; Figure S7A,B). Similarly, the self‐connectivity of the MFG.R correlated with HAMD‐17 score reductions (*r* = 0.44, *p* = 0.034; Figure S7C). Notably, the connectivity from IPL.R to MFG.R, as well as the self‐connectivity of the AMYG.L also exhibited positive relationships with BSI‐CV improvements (*r* = 0.46, *p* = 0.031; *r* = 0.45, *p* = 0.027; Figure S8A,B), suggesting higher baseline ECs corresponding to better anti‐suicidal ideation efficacy. Whereas, the connectivity from the ACC.R to ACC.L and the self‐connectivity of ACC.R were negatively correlated with BSI‐CV scores, indicating that possibly, certain connectivity patterns may be associated with better TMS anti‐suicidal efficacy (*r* = −0.41, *p* = 0.049; *r* = −0.45, *p* = 0.028; Figure S8C,D).

No significant correlations between the abnormal FC and depression and suicidality reduction scores existed.

## 4 Discussion

The present study focuses on the anomalies in the CCN‐AN circuitry in MDD based on a multisite dataset, that is, REST‐meta‐MDD, and offers a dependable and objective biomarker for MDD through a novel classifier. More importantly, the observed aberrant ECs of CCN and AN were found to be associated with depression severity and closely correlated with depression and suicidal ideation alleviation after TMS treatment, giving the implication that these locations could perhaps be efficacious targets for future interventions.

Numerous studies have utilized SVM or other machine learning (ML) algorithms to identify an objective biomarker for MDD diagnosis [42, 43, 44, 45]. Classification accuracies varied from less than 60% all the way to as high as 99% [46]. For example, Zeng and colleagues constructed a classifier based on resting state functional connectivity using unsupervised machine learning, achieving a classification score of 92.5% [47]. Conversely, another study that utilized functional connectivity as features reported a lower accuracy of 69.6% [48]. Up until now, many studies have been limited by small sample sizes and are too confined to specific cohorts. Attempts to generalize classifications across independent datasets, as demonstrated by Sundermann et al. [49], have only yielded results similar to chance [49]. Recently, Gallo and colleagues employed whole‐brain functional connectivity to distinguish MDD from HC based on REST‐meta‐MDD and PsyMRI consortia, achieving a mean classification accuracy of 61% [44]. In our study, we focused solely on the EC of the AN and CCN, resulting in a classification accuracy score of 79.1% based on five‐fold validation and 75% based on LOSOCV. The acceptable classifier performance, demonstrated across 13 independent sites, indicates the potential to construct an MDD diagnosis model by detecting abnormalities in the CCN and AN that can be generalized across diverse cohorts. Despite the potential effects of heterogeneity on the fair classification performance, it does provide a lower bound of the classification for the current classification model.

Overall, EC was more sensitive to abnormalities in the CCN‐AN circuit of MDD compared with FC. Our current study utilized both functional and effective connectivity as the basic measures. Instead of functional connectivity characterizing the interactions between two units without directional information, effective connectivity characterizes the causal influences from one unit to another. The signal direction is crucial for revealing the pathological mechanisms of various disorders and treatment effects [24]. When compared to FC, a previous study revealed EC could be more effective in detecting abnormalities of MDD and distinguishing MDD from HCs [25]. In our current study, FC only detected significant differences in self‐connectivity of MFG.R between MDDs and HCs, whereas EC showed more sensitivity to discrepancies in the CCN‐AN circuit between MDD and the HCs. More importantly, these aberrant ECs were closely correlated with depression and suicidal scale reductions (Figures S7 and S8), indicating the predictive power of EC and the potential for TMS treatment efficacy.

The CCN‐AN circuit is crucial for the proper functioning of MDD patients. Dysregulation within this circuit significantly impacts inefficient emotion regulation. Indeed, numerous studies have demonstrated that impaired connectivity between the brain regions within the CCN—such as the DLPFC, MOFC—and those within the AN—particularly AMYG (a central node of AN)—plays a significant role in emotional processing deficits observed in MDD patients [7, 22, 50]. Aligning with this previous research, our current study detected decreased connectivity from the CCN to AN, with increased connectivity from AN to CCN for MDD (Figure 2). Moreover, the altered connectivity from IPL (one node of CCN) to AMYG (one node of AN) negatively correlated with depression scores (Figure 3D), and the self‐connectivity of brain areas within AN and CCN, including the ACC, AMYG, MOFC, MFG, and IPL, positively correlated with HAMD scores (Figure S6). All these findings suggest both bottom‐up and top‐down regulation of MDD, which collectively contribute to the disrupted mood regulation states observed in MDD.

It is worth noting that the alterations observed in connectivity within the CCN‐AN circuit were closely correlated with depression and suicidal alleviation after TMS treatment according to the Stanford Neuronavigational Therapy (SNT) procedure. The ACC stands as an important biomarker in the pathology of MDD and has also been underscored for its significance by numerous studies [51, 52, 53]. The ACC of MDD patients presented hyperactivation when performing affective processing tasks [54], and showed increased voxel‐based pathophysiological metrics (regional homogeneity, and amplitude of low frequency fluctuation) [11] demonstrated by meta‐analysis. Consistent with previous studies, the rostral ACC also plays an important role as a deep effective target for TMS therapy in MDD [21, 55, 56, 57]. The self‐connectivity of the ACC is closely correlated with depression scores (as measured by HAMD‐17 and MADRS; Figure S6) which further demonstrate the significance of the ACC for TMS therapy in MDD.

The AMYG stands as another central hub of the AN, and also plays an important role in the pathology of MDD [7, 23, 58, 59]. Alterations of the AMYG‐related circuit—specifically, the connectivity between the AMYG and the DLPFC, VPFC, MOPFC, ACC—are correlated with the dysfunction of emotion regulation in MDD [7, 59, 60, 61]. Furthermore, studies found that MDD patients presented significantly decreased connectivity between the AMYG and subgenual ACC [62, 63], which is in line with our findings regarding the reduced influence from the ACC.R to the AMYG.R. Furthermore, the disrupted connectivity of the IPL‐AMYG complex is negatively correlated with depression score reductions (Figure 4C). Furthermore, the self‐connectivity of the AMYG is closely correlated with depression severity and suicidality alleviation (Figures S7 and S8) which further indicates that AMYG might be another important effective TMS target for alleviating depression.

Last but not least, the connectivity of IPL‐ACC and IPL‐AMYG closely correlates with depression and suicidal ideation (BSI‐CV) reductions (Figure 4). This might suggest that IPL might be another potential superficial stimulation target for TMS therapy in the future.

Here, we will state some limitations that exist in our study. First, the large multi‐dataset of REST‐meta‐MDD with large heterogeneity consisted of widely ranging demographic information, clinical subtypes, and scanning parameters, which presumably reduced the classification accuracy and our ability to maintain strict control over inclusion criteria. Moreover, the selection of ROIs is confined to the AAL template, which is not the end‐all be‐all of brain regions and only serves as a guide for classification. Additionally, due to the intricacy of MDD and its large heterogeneity of different subtypes, more information needs to be collected regarding bio‐chemical, genomic, anatomical, neuroimaging, and behavioral aspects of MDD. And finally, validating the predictive power of our identified biomarkers to other treatments, including antidepressant drugs and psychotherapies, is critical and should be prioritized in future investigations to ensure a comprehensive understanding of anti‐depression neural mechanisms for diverse therapeutic approaches. All of these topics need to slowly be integrated into a holistic system for detecting the abnormalities and potential biomarkers to help us define and diagnose MDD.

## 5 Conclusions

In conclusion, EC is a more sensitive and reliable measure in detecting abnormalities in MDD. Disturbances in the circuitry of CCN‐AN in regards to MDD based on a comprehensive multi‐site dataset suggest that the disrupted top‐down and bottom‐up regulation systems play an important role in MDD. Notably, these abnormalities, particularly the connectivity between the IPL and the ACC, as well as the IPL and the AMYG, were closely correlated with depression and suicidal alleviation in an independent dataset treated with neuronavigational TMS therapy. These findings help to pave the way for predictive and analytical methods using biomarkers based on mass data rather than specific cohorts or on a case‐by‐case basis. Overall, this research offers fresh perspectives on the neural underpinnings of MDD, enhancing MDD diagnosis and facilitating precision‐targeted interventions.

## Conflicts of Interest

The authors declare no conflicts of interest.

## Supporting information


Appendix S1.

## Data Availability

Dataset‐1 is available on REST‐meta‐MDD Consortium; Dataset‐2 is available from the corresponding author.
