# Supplementary material for: Disruptions in Cognitive‐Affective Circuitry in Major Depression Disorder: Insights From REST‐Meta‐MDD and Its Implication for Predicting TMS Treatment Efficacy
Source: CNS Neurosci Ther. 2025 Aug 4;31(8):e70533. doi: 10.1111/cns.70533 (PMC12319422; doi:10.1111/cns.70533)
Supplement: Supplementary file 1 — Appendix S1. [file CNS-31-e70533-s001.docx]

## Supplementary materials

Table S1. The sensitivity, specificity, classification accuracy, and AUC score of each fold.

| Fold | 0 | 1 | 2 | 3 | 4 | Mean | SD |
| --- | --- | --- | --- | --- | --- | --- | --- |
| Sensitivity (%) | 75.3 | 77.4 | 80.6 | 81.7 | 76.3 | 78.3 | 2.8 |
| Specificity (%) | 80.9 | 77.5 | 78.7 | 76.4 | 80.9 | 78.9 | 2.0 |
| Accuracy (%) | 78.0 | 77.5 | 79.7 | 79.1 | 78.6 | 78.6 | 0.9 |
| AUC | 0.87 | 0.86 | 0.87 | 0.86 | 0.87 | 0.87 | 0.01 |

AUC: area under ROC curve; ROC: receiver operating characteristic. SD: standard deviation

Table S2. The sensitivity, specificity, classification accuracy, and AUC score of each site.

|  | S1 | S2 | S3 | S4 | S5 | S6 | S7 | S8 | S9 | S10 | S11 | S12 | S13 | Mean | SD |
| --- | --- | --- | --- | --- | --- | --- | --- | --- | --- | --- | --- | --- | --- | --- | --- |
| Sensitivity (%) | 78.6 | 78.3 | 80 | 86.2 | 66.7 | 89.5 | 80.6 | 78.7 | 83.7 | 70 | 90.5 | 53.8 | 71.4 | 77.5 | 10.1 |
| Specificity (%) | 62.9 | 81.3 | 87.5 | 74.4 | 66.7 | 93.8 | 82.5 | 80.4 | 72.2 | 50 | 47.1 | 62.5 | 76.9 | 72.2 | 13.9 |
| Accuracy (%) | 69.8 | 79.5 | 84.6 | 80.6 | 66.7 | 91.4 | 81.6 | 79.6 | 78.5 | 59.1 | 71.1 | 58.6 | 74.1 | 75.0 | 9.7 |
| AUC | 0.75 | 0.87 | 0.9 | 0.84 | 0.76 | 0.95 | 0.89 | 0.85 | 0.89 | 0.73 | 0.89 | 0.67 | 0.8 | 0.83 | 0.08 |

AUC: area under ROC curve; ROC: receiver operating characteristic. SD: standard deviation


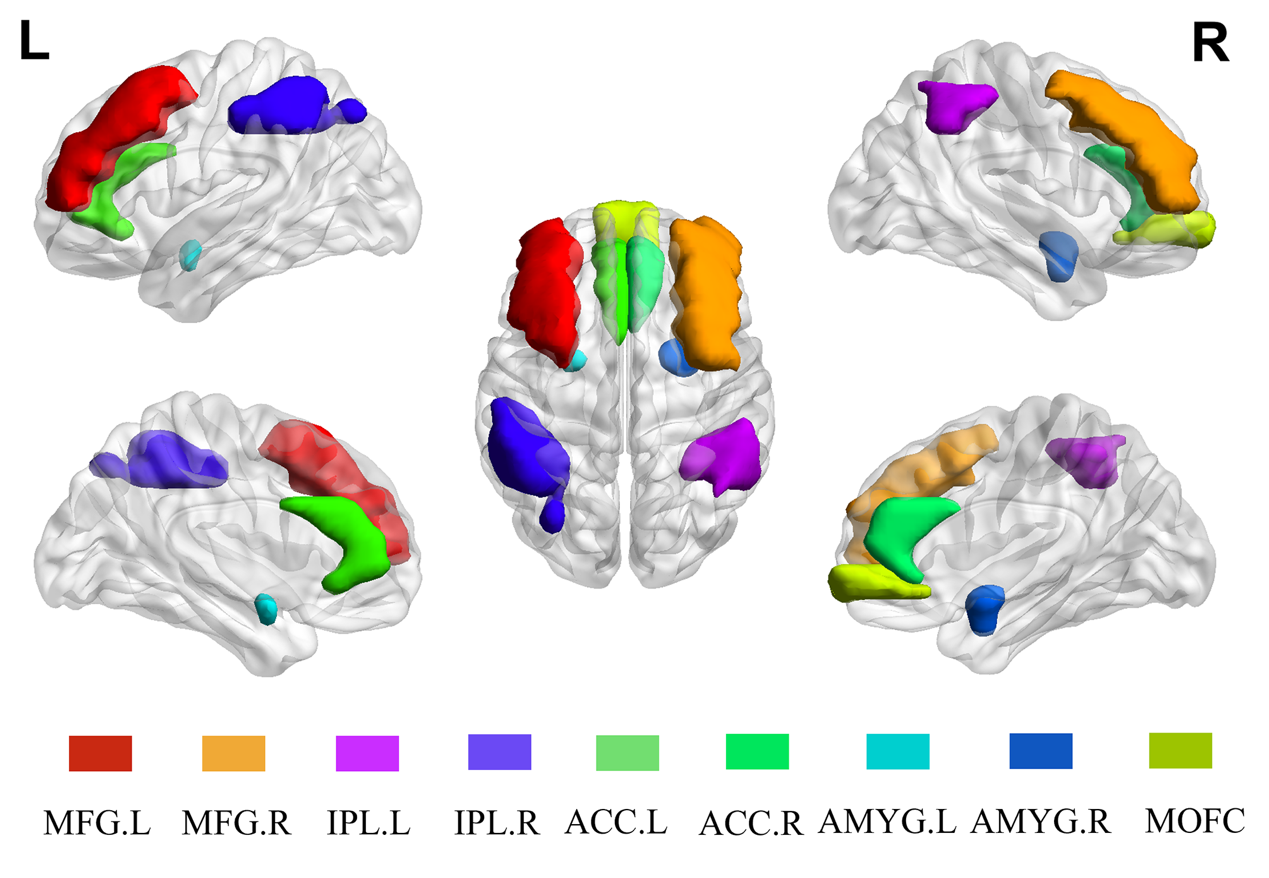


Figure S1. The ROIs selected in CCN and AN. CCN: cognitve control network; AN: affective network.


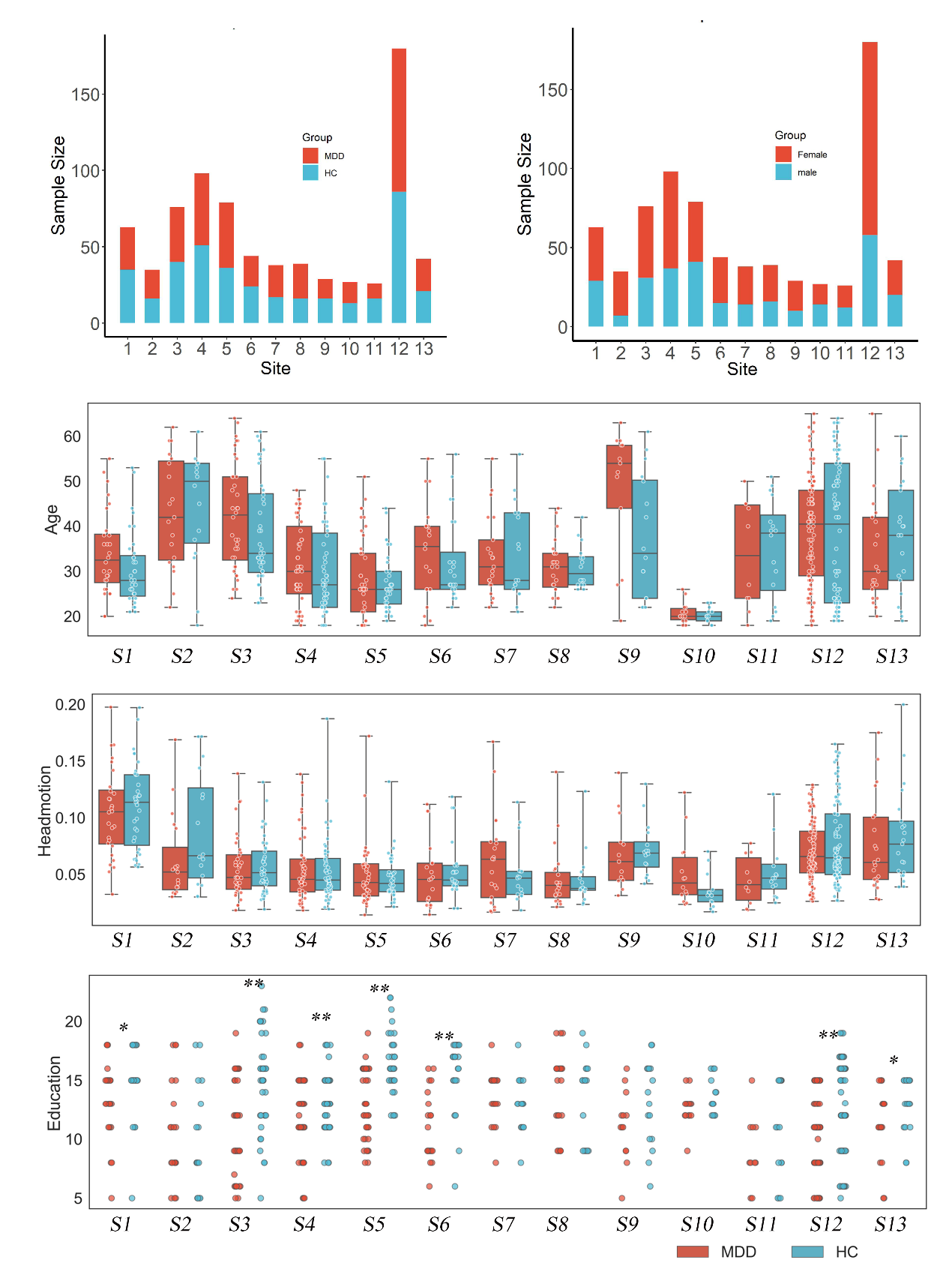


Figure S2. The basic information of included participants in Leave-One-Site-Out Cross Validation. *: *p* < 0.05; **: *p* < 0.01.


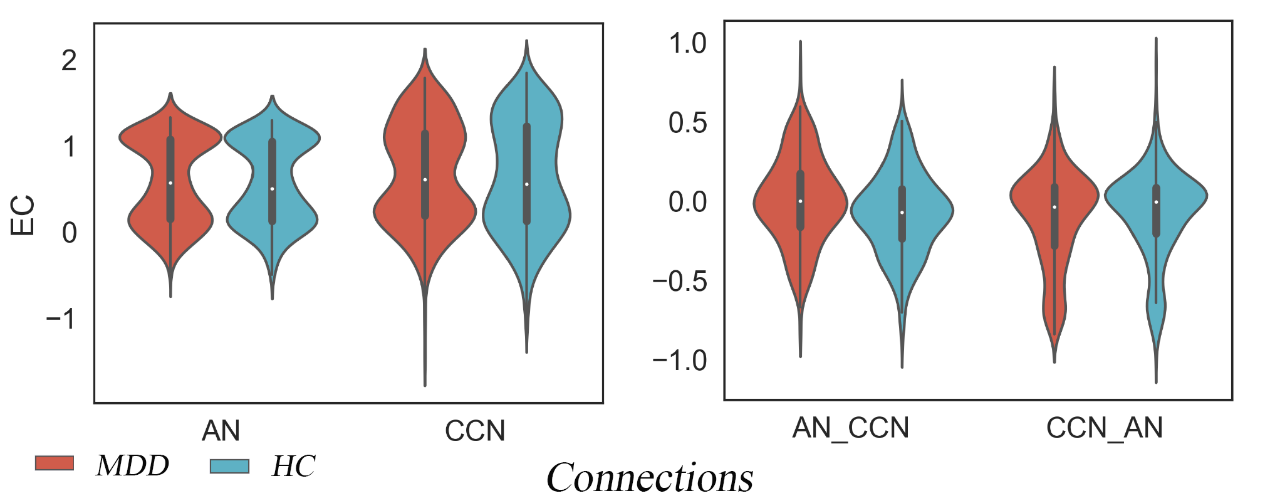


Figure S3. The ECs within the AN and CCN, and between the two networks. EC: effective connectivity; AN: affective network; CCN: cognitive control network. Significant interaction (F = 17.051, df =1, *p* < 0.001) observed in the connections between AN and CCN circuit, i.e., The connections from the CCN to AN decreased, while those from AN to CCN increased for MDDs when compared with HCs.


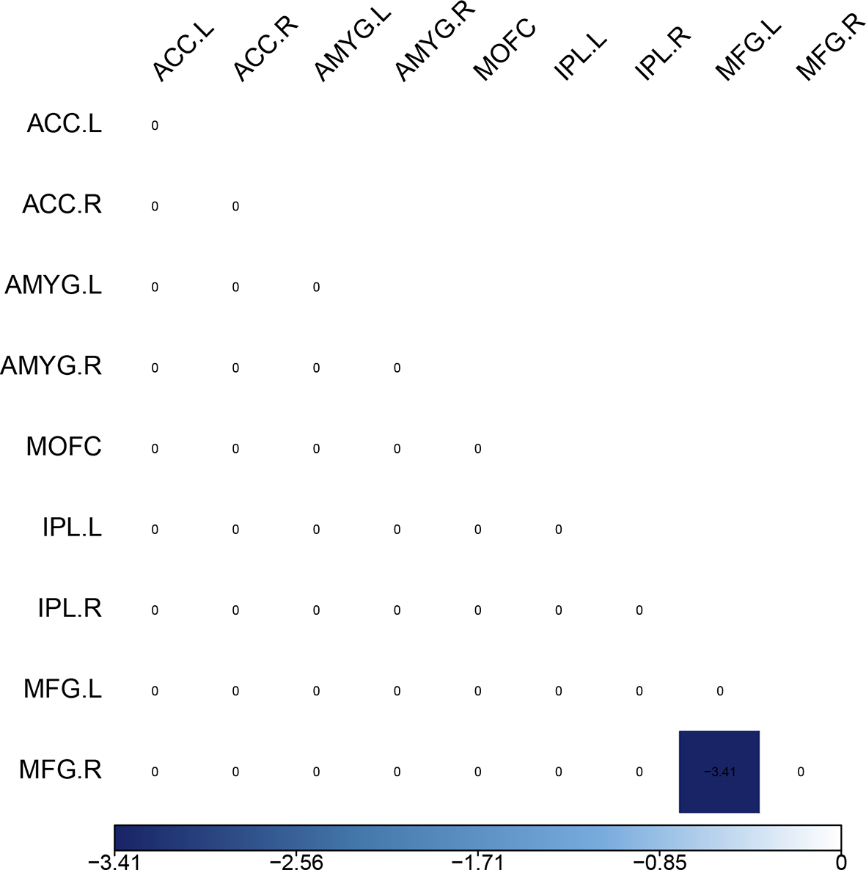


Figure S4. Significant different functional connectivity between MDD and HC (FDR correction, *q* < 0.05). Negative value represents significant lower connectivity for MDD compared with HC.


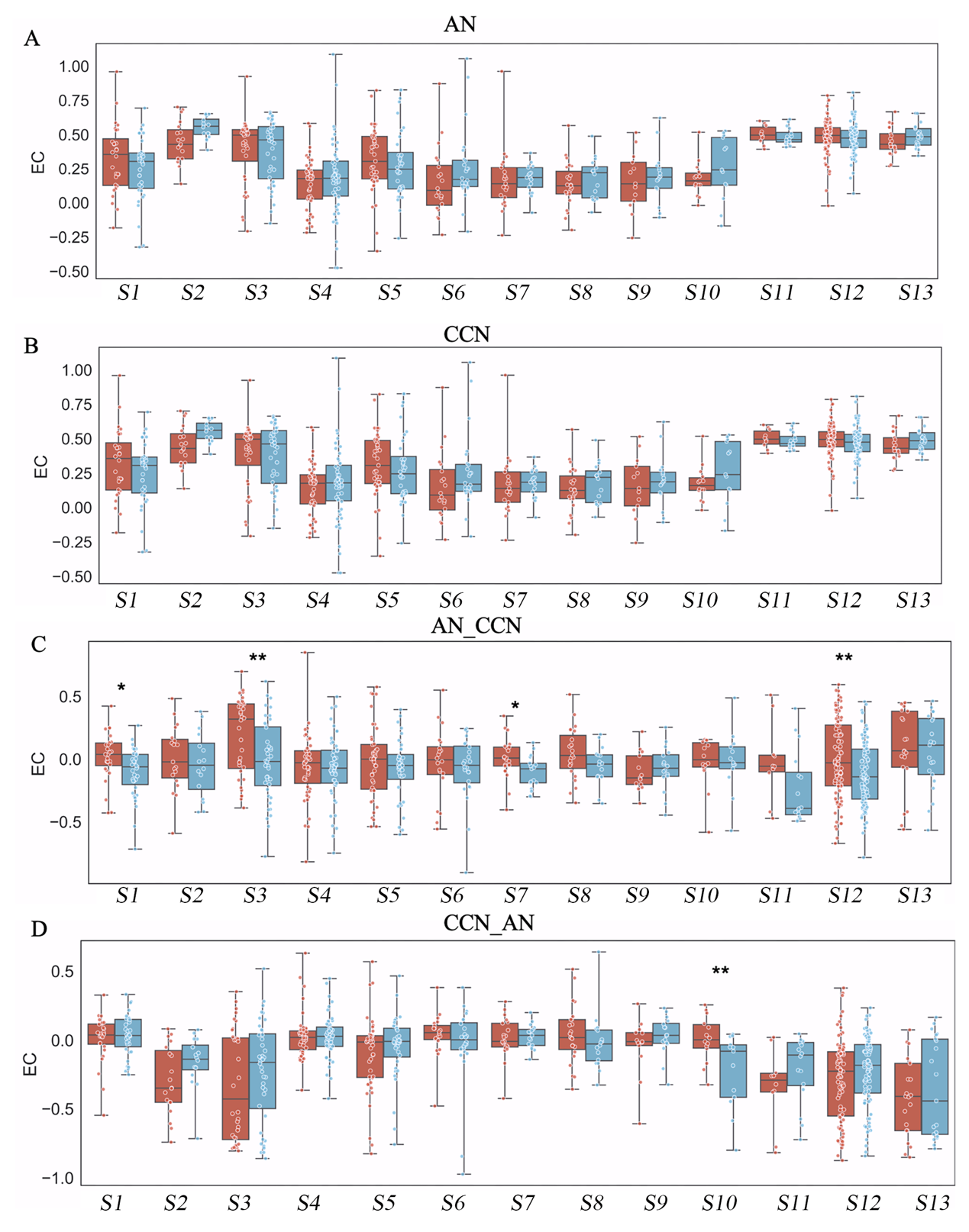


Figure S5. The ECs within the AN and CCN, and between the two networks of 13 independent data sites; EC: effective connectivity; AN: affective network; CCN: cognitive control network. *: *p* < 0.05; **: *p* < 0.01.


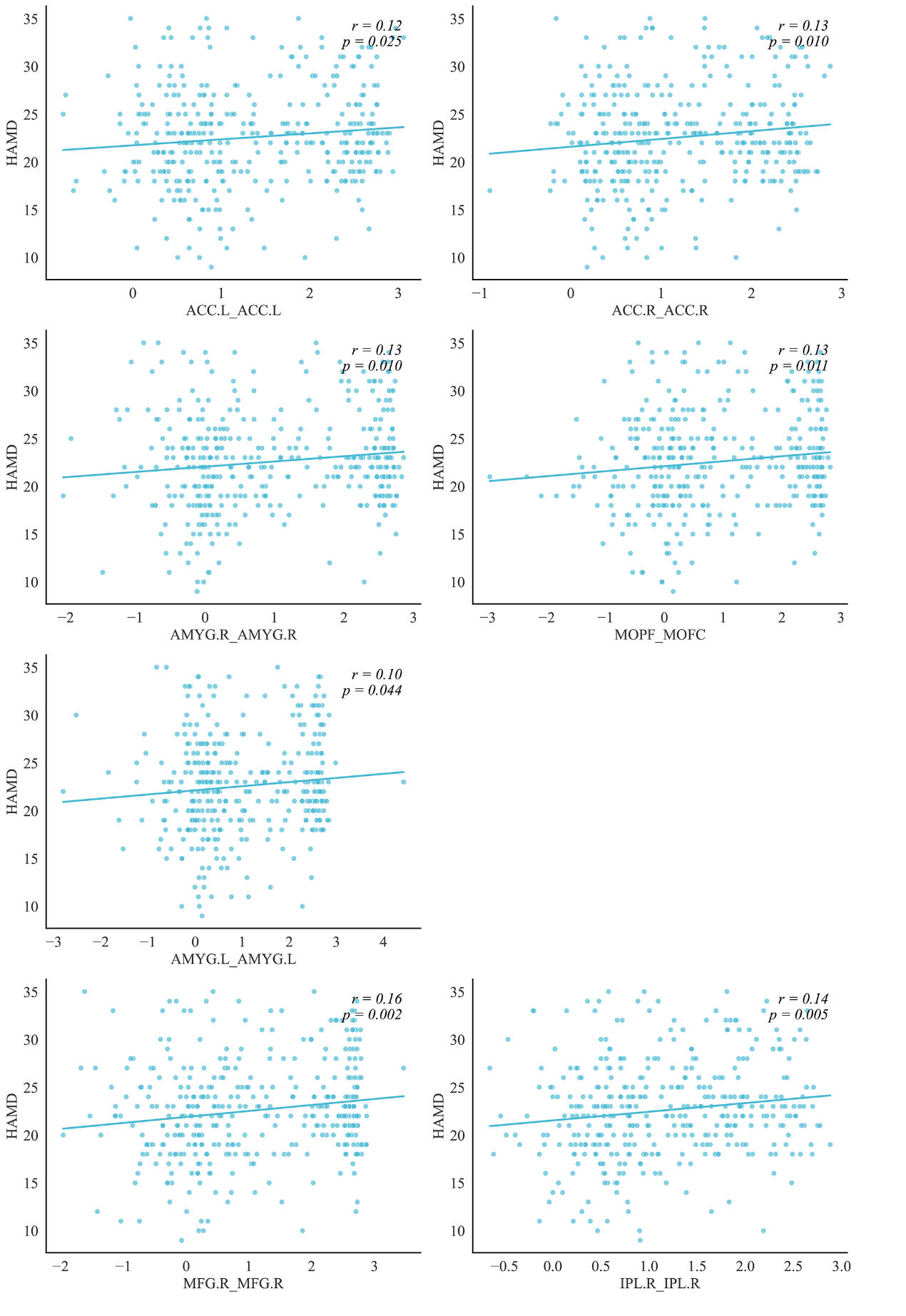


Figure S6. The correlations between the ECs and depression scores. HAMD: Hamilton Depression Rating Scale; ACC: the anterior cingulate cortex; MOFC: medial orbitofrontal cortex; AMYG: amygdala; MFG: middle frontal gyrus; IPL: the inferior parietal lobule.


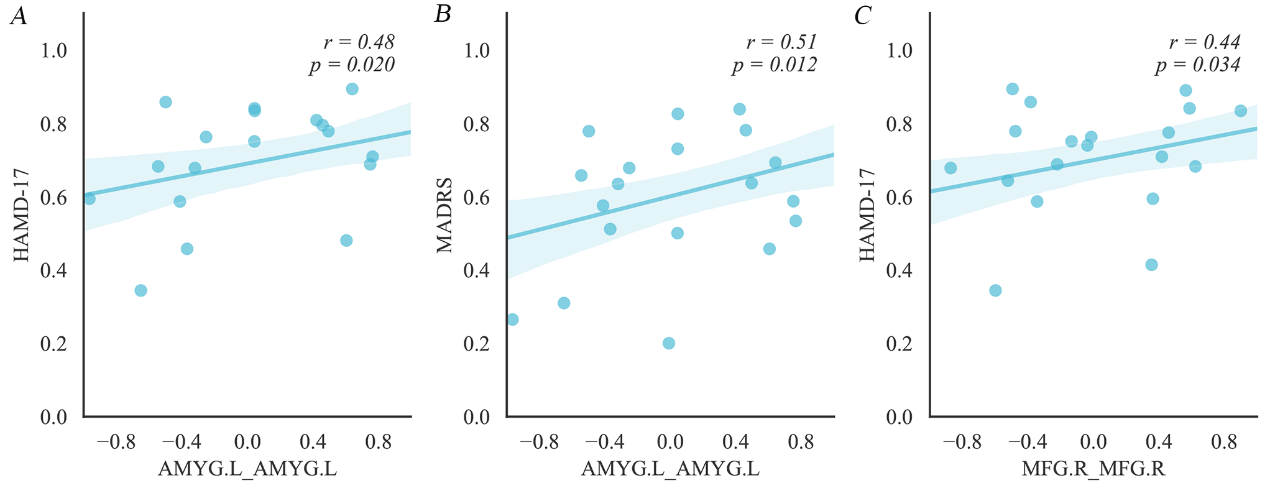


Figure S7. The predictive power of the abnormal connections within AN or and CCN for TMS antidepressant efficacy. The correlations between the effective connectivity of AMYG.L-AMYG.L (A, B), MFG.R-MFG.R (C) and depression score reductions; HAMD: the 17-item Hamilton Depression Rating Scale; MADRS: Montgomery-Asberg Depression Rating Scale. Responder: those patients with the reduction of depression scores larger than 50%; Non-responder: those patients with the reduction of depression scores less than 50%.


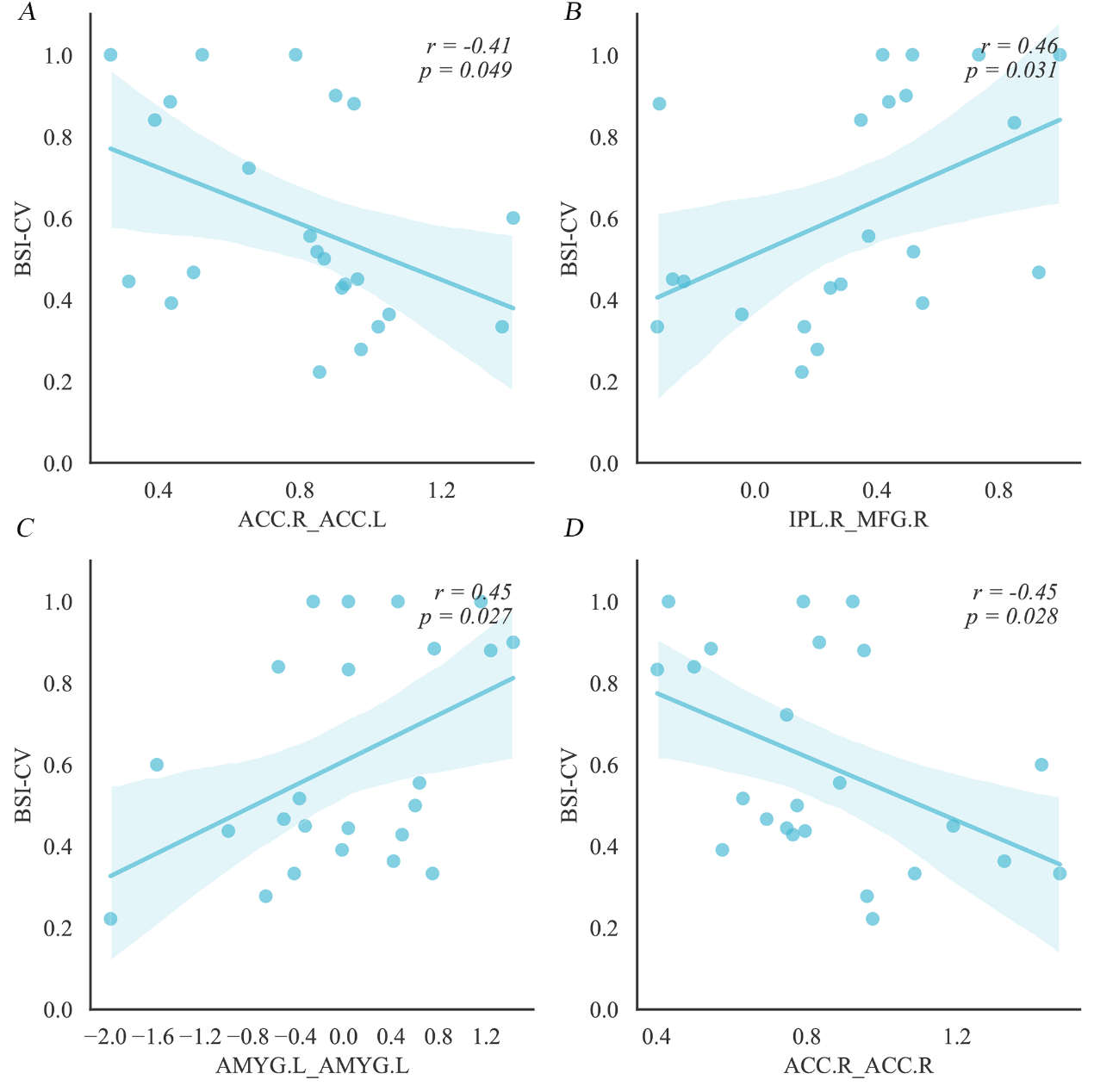


Figure S8. The predictive power of the abnormal connections within AN or and CCN for TMS anti-suicidal efficacy. The correlations between the effective connectivity of IPL.R-MFG.R (A), AMYG.R-AMYG.L (B), ACC.R-ACC.L (C), ACC.R-ACC.R (D) and suicidality alleviation; BSI-CV: Beck Suicide Intentionality Scale - Chinese Version; ACC: the anterior cingulate cortex; AMYG: amygdala; IPL: inferior parietal lobule; MFG: middle frontal gyrus; Responder: those patients with the reduction of BSI-CV scores larger than 50%; Non-responder: those patients with the reduction of BSI-CV scores less than 50%.
